# Chronic Kidney Disease‐Associated Pruritus in Haemodialysis: A Mixed‐Methods Study of Symptom Burden and Patient Experience

**DOI:** 10.1111/jorc.70075

**Published:** 2026-07-29

**Authors:** Gülay Turgay, Çiğdem Özdemir Eler

**Affiliations:** ^1^ Dialysis Programme, Vocational School of Health Services Başkent University Ankara Turkey

**Keywords:** chronic kidney disease‐associated pruritus, haemodialysis, mixed methods research, nursing care, symptom burden

## Abstract

**Background:**

Chronic kidney disease‐associated pruritus is a common and often under‐recognized symptom among people receiving haemodialysis and may substantially impair daily life and wellbeing.

**Objectives:**

To determine the prevalence and severity of pruritus in people receiving haemodialysis and to explore the lived experiences of those with moderate‐to‐severe pruritus.

**Design:**

Explanatory sequential mixed‐methods study.

**Participants:**

A total of 294 adults receiving haemodialysis in three dialysis centres participated in the quantitative phase. The qualitative phase included 48 participants selected from the quantitative sample who had moderate‐to‐severe pruritus, defined as a Five‐D Itch Scale score of 12 or higher.

**Measurements:**

Data were collected between October 2025 and January 2026. Quantitative data were collected using the Five‐Dimensional Itch Scale and a sociodemographic and clinical information form developed for this study. Qualitative data were generated through at least two semi‐structured interviews with each participant and analysed using reflexive thematic analysis. Integration was performed at the interpretation stage.

**Results:**

The mean Five‐Dimensional Itch Scale score was 8.00 ± 3.21, and moderate‐to‐severe pruritus prevalence was 16.3%. Multivariable analysis showed lower symptom reporting among married participants, whereas comorbidity and previous pruritus‐related education were associated with higher symptom reporting. Qualitative findings indicated that pruritus was experienced as a fluctuating burden affecting sleep, daily comfort, and social roles. Participants described trial‐and‐error self‐management strategies and expressed a need for proactive assessment, practical guidance and individualised nursing care.

**Conclusions:**

Chronic kidney disease‐associated pruritus remains a multidimensional burden in haemodialysis care. Standardised assessment and person‐centred nursing care may improve its recognition and management.

## 1 Introduction

Chronic kidney disease‐associated pruritus is a common and distressing symptom in people receiving haemodialysis and can substantially compromise quality of life, particularly by disrupting sleep, daily functioning and overall wellbeing (Mettang and Kremer 2015). Although attention to symptom burden in kidney care has increased, pruritus may still be insufficiently recognised in routine practice, which can limit timely and effective management (Jha et al. 2022).

Chronic kidney disease‐associated pruritus should be examined not only in terms of prevalence and severity but also through patients' lived experiences of the symptom in everyday life. Therefore, this study aimed to determine the prevalence and severity of pruritus in people receiving haemodialysis and to explore the lived experiences of those with moderate‐to‐severe pruritus.

## 2 Literature Review

Current evidence suggests that chronic kidney disease‐associated pruritus is a multidimensional symptom burden and is associated with sleep disturbance, functional limitations in daily activities, reduced psychological wellbeing, and poorer health‐related quality of life (Wang et al. 2025; Latus et al. 2025). However, reported prevalence varies depending on the assessment instrument and the threshold used to define severity; recent clinical syntheses suggest that moderate‐to‐severe pruritus may affect approximately 31%–40% of people receiving haemodialysis (Latus et al. 2025). In a prospective multicentre observational study, systematic screening identified a prevalence of 23.5% for moderate‐to‐very severe pruritus, and a substantial proportion of affected individuals had not been recognised before routine screening was introduced (Lanot et al. 2023). These findings indicate that the visibility and management of pruritus may depend on systematic screening and regular symptom assessment during routine follow‐up (Lanot et al. 2023; Liossatou et al. 2025).

Similarly, analyses from the multinational Dialysis Outcomes and Practice Patterns Study suggest that pruritus burden may be underestimated within haemodialysis units, indicating that the symptom can be overlooked in everyday care when structured assessment and consistent follow‐up are not in place (Rayner et al. 2017). These findings support the need for systematic symptom screening and monitoring as part of person‐centred kidney care (Rayner et al. 2017; Liossatou et al. 2025). In addition, skin‐directed supportive approaches have been reported to contribute to symptom relief in this population. For example, topical vitamin D has been associated with improvement in chronic kidney disease‐associated pruritus, and moisturiser treatment has been shown to reduce pruritus accompanied by xerosis in patients undergoing dialysis (Jung et al. 2015; Yoshida et al. 2021). Together, these findings suggest that routine care should focus not only on recognising pruritus but also on practical, feasible nursing care strategies that address the skin‐related dimension of the symptom.

Effective pruritus management depends on multidimensional and clinically robust assessment tools. The Five‐D Itch Scale is a self‐reported instrument that evaluates the duration, severity, direction, distribution, and impact of pruritus on daily activities (Elman et al. 2010). The Turkish version has demonstrated validity and reliability and may support symptom monitoring and nursing care planning in kidney care settings (Akyar and Altınok Ersoy 2018). However, while quantitative measures are essential for describing symptom burden, they may not fully explain how pruritus is experienced in everyday life, how symptom fluctuations are interpreted, or why individuals adopt particular coping and self‐management strategies. Patient‐experience studies also show that chronic kidney disease‐associated pruritus may be perceived as an intrusive and persistent symptom affecting sleep, mood, social participation and everyday coping, which supports the need to examine patients' accounts alongside quantitative severity scores (Menzaghi et al. 2023; Huang et al. 2024). Qualitative approaches can therefore provide complementary insights that deepen clinical interpretation (Huang et al. 2024; Tarp et al. 2017).

Despite the expanding literature on prevalence and symptom burden, studies that bring together quantified pruritus burden with patients' accounts of lived experience remain limited in haemodialysis care. When systematic screening is not embedded in care processes, pruritus burden may remain insufficiently visible in routine practice, and quantitative findings alone may be inadequate to explain the day‐to‐day impact of pruritus and the coping patterns patients develop. Therefore, this study used an explanatory sequential mixed‐methods design to determine the prevalence and severity of pruritus in 294 people receiving haemodialysis using the Five‐D Itch Scale and to explore the lived experiences of 48 participants with moderate‐to‐severe pruritus, defined as a Five‐D score of 12 or higher, through in‐depth interviews.

## 3 Materials and Methods

### 3.1 Design

This study employed an explanatory sequential mixed methods design to examine pruritus multidimensionally in people receiving haemodialysis (Creswell and Plano Clark 2018). In the first phase, a cross‐sectional quantitative design was used to assess the prevalence and severity of pruritus and its impact on daily life. In the second phase, semi‐structured in‐depth interviews were conducted within a descriptive qualitative design with 48 purposively selected participants from the quantitative sample who had moderate‐to‐severe pruritus, defined as a Five‐D Itch Scale total score of 12 or higher (Sandelowski 2000). A maximum variation approach was used to capture diversity in participant characteristics and experiences.

### 3.2 Setting

The study was conducted in three dialysis centres affiliated with Başkent University Ankara Hospital. Across these centres, approximately 520 patients were receiving maintenance haemodialysis. The units operated 110 haemodialysis machines and provided treatment 6 days a week (Monday‐Wednesday‐Friday and Tuesday‐Thursday‐Saturday) in two daily shifts, morning and midday. This setting provided access to a large haemodialysis population for the quantitative phase and supported the recruitment of a qualitatively diverse sample with varying sociodemographic and clinical characteristics.

### 3.3 Population and Sample

#### 3.3.1 Quantitative Phase

The quantitative population comprised 520 adult patients receiving haemodialysis in the three study centres. Rather than drawing a sample, the study aimed to include the whole population. Patients who met the eligibility criteria and provided written informed consent formed the quantitative sample (*n* = 294).

Inclusion criteria

- Aged 18 years or older
- Receiving maintenance haemodialysis for at least 6 months
- Able to read and write Turkish and communicate verbally

Exclusion criteria

- A documented psychiatric diagnosis in the medical record or a current clinically evident psychiatric condition that could compromise reliable self‐reporting, informed participation or completion of the qualitative interview.
- A known active dermatological condition that could cause pruritus (e.g., psoriasis, atopic dermatitis)
- Cholestatic liver disease or another serious systemic condition associated with pruritus
- Acute infection, active malignancy, or another acute medical condition likely to explain pruritus
- Initiation of a new systemic antipruritic treatment within the previous month

### 3.4 Qualitative Phase

The qualitative sampling frame consisted of participants from the quantitative phase whose pruritus severity had been assessed using the 5‐D Itch Scale and who had moderate‐to‐severe pruritus. After completion of the quantitative phase, participants from the quantitative sample with a 5‐D Itch Scale total score of 12 or higher were classified as having moderate‐to‐severe pruritus, based on the categorisation proposed by Lai et al. (2017).

Purposive sampling was used, with a maximum variation strategy to ensure diversity in age, sex, dialysis vintage, employment status, and duration of pruritus. Data collection continued until no new themes or meaningful insights relevant to the research questions were identified, consistent with the principle of data saturation, resulting in a final qualitative sample of 48 participants (Braun and Clarke 2006, 2021; Sandelowski 2000). All 48 participants invited to the qualitative phase agreed to participate and completed the interview process.

### 3.5 Data Collection Instruments

#### 3.5.1 Sociodemographic and Clinical Information Form

The Sociodemographic and Clinical Information Form was an ad hoc form developed by the researchers for this study. It was prepared based on the literature and clinical expert input to collect sociodemographic, clinical and treatment‐related variables relevant to chronic kidney disease‐associated pruritus in haemodialysis care (Rigatto et al. 2024; Latus et al. 2025; Sukul et al. 2021). The form included age, sex, marital status, educational level, employment status, dialysis vintage, weekly haemodialysis session frequency, comorbidities, vascular access type, pruritus history and previous education related to pruritus. The form was reviewed by two academic experts for content and clarity and was pilot‐tested with 10 people receiving haemodialysis; no item modifications were required after the pilot.

### 3.6 5‐D Itch Scale

Pruritus severity and its impact on daily life were assessed using the 5‐D Itch Scale, developed by Elman et al. (2010). The scale includes five domains‐duration, degree, direction, disability and distribution‐each scored from 1 to 5, yielding a total score from 5 to 25, with higher scores indicating more severe pruritus (Elman et al. 2010). The Turkish validity and reliability study of the scale was conducted by Akyar and Altınok Ersoy (2018) in patients with chronic kidney disease. The Turkish version was reported to be suitable for this population and demonstrated acceptable reliability and validity. In this study, the Turkish version was used. Pruritus severity was interpreted according to the categorisation proposed by Lai et al. (2017): ≤ 8 no pruritus, 9–11 mild, 12–17 moderate, 18–21 severe and ≥ 22 very severe. Because total 5‐D Itch Scale scores could include decimal values, scores of 11.5 and above were rounded up to 12 for categorical classification. For quantitative analyses, participants with a total score of 12 or higher were classified as having moderate‐to‐severe pruritus. In the present study, the internal consistency of the 5‐D Itch Scale was acceptable (Cronbach's *α* = 0.77).

### 3.7 Qualitative Interview Guide

A semi‐structured interview guide was developed by the researchers based on the relevant literature (Liossatou et al. 2025; McKie et al. 2023), the study aims, and the quantitative findings (Sandelowski 2000; Braun and Clarke 2006). The guide included open‐ended questions covering: (1) the clinical and emotional experience of pruritus, (2) its effects on daily life, sleep, and social relationships, (3) coping and self‐management strategies, (4) experiences with haemodialysis nurses and other health professionals, and (5) expectations regarding pruritus management.

Before qualitative data collection began, the guide was pilot‐tested with 10 people receiving haemodialysis to assess clarity and feasibility. No item modifications were required following pilot testing. Pilot interviews were not included in the main qualitative dataset. The interview guide is provided in Supporting Information S1: Appendix 1.

### 3.8 Data Collection Procedure

Data collection took place between October 2025 and January 2026. Quantitative data were collected on haemodialysis days when participants were clinically stable, through face‐to‐face interviews conducted by a member of the research team. Participants were provided with verbal and written information about the purpose, scope, and procedures of the study. Those who gave written informed consent completed the Sociodemographic and Clinical Information Form and the 5‐D Itch Scale. Completing the questionnaires took approximately 10–15 min, and short breaks were offered if participants experienced fatigue or discomfort.

Following completion of the quantitative phase, participants from the quantitative sample with a 5‐D Itch Scale total score of 12 or higher were identified and invited to take part in the qualitative phase. All invited participants (*n* = 48) agreed to participate and completed the qualitative data collection process. Interviews were conducted by the researcher in a quiet, private education room near the dialysis unit. There was no pre‐existing therapeutic or clinical relationship between the interviewer and the participants, and no third person was present during the interviews.

To allow for more in‐depth exploration and clarification of participants' experiences, each participant took part in at least two semi‐structured interviews conducted approximately 1 week apart. The first interview focused on participants' experiences of pruritus, its effects on daily life and their coping strategies. Follow‐up interviews were conducted to clarify and deepen issues raised in the first interview and to obtain richer accounts of participants' care experiences and expectations. With participants' permission, all interviews were audio‐recorded. The first interviews lasted a mean of 35 min, and follow‐up interviews lasted approximately 20–25 min. Field notes were made during and immediately after the interviews to capture contextual observations and initial analytic reflections.

### 3.9 Data Analysis

#### 3.9.1 Quantitative Data Analysis

Quantitative data were analysed using IBM SPSS Statistics 25. Descriptive statistics were presented as frequencies, percentages, means with standard deviations, medians, and minimum‐maximum values. Total 5‐D Itch Scale scores were categorised according to the classification proposed by Lai et al. (2017). For inferential analyses, pruritus severity was further dichotomised as no/mild pruritus (5‐D < 12) and moderate‐to‐severe pruritus (5‐D ≥ 12).

Associations between categorical variables and moderate‐to‐severe pruritus were examined using the chi‐square test. To identify independent factors associated with moderate‐to‐severe pruritus, multivariable binary logistic regression analysis was performed. Variables were entered into the model based on the literature and the results of univariable analyses (*p* < 0.20). Findings are reported as odds ratios with 95% confidence intervals.

Model fit was evaluated using the Omnibus test of model coefficients and the Hosmer–Lemeshow goodness‐of‐fit test, while explanatory power was assessed using Nagelkerke *R* ^2^. A two‐sided *p* value of < 0.05 was considered statistically significant.

### 3.10 Qualitative Data Analysis

Qualitative data were analysed using Braun and Clarke's reflexive thematic analysis following verbatim transcription of the audio‐recorded interviews (Braun and Clarke 2006, 2021). The analysis was iterative and focused on developing an in‐depth interpretation of meaning patterns relevant to the quantitative findings. Reporting of the qualitative component was informed by the Consolidated Criteria for Reporting Qualitative Research for interview‐based qualitative studies (Tong et al. 2007).

The analysis involved the following stages:

1. Familiarisation with the data: The researchers read and re‐read the transcripts to become closely familiar with the data, while noting initial impressions and salient points. Field notes were reviewed alongside the transcripts to support contextual interpretation.
2. Generating initial codes: The transcripts were examined line by line, and initial data‐driven codes were generated from meaningful units of text.
3. Constructing candidate themes: Conceptually related codes were grouped to form candidate themes and subthemes.
4. Reviewing themes: Themes were reviewed against the dataset to assess coherence and distinctiveness; overlapping themes were merged, and weaker themes were removed or restructured.
5. Defining and naming themes: The scope and boundaries of each theme were clarified, analytic definitions were developed, and themes were named.
6. Interpretation and integration: Themes were interpreted in relation to the research questions and the quantitative findings, with particular attention to how the experiences of participants with moderate‐to‐severe pruritus helped explain the quantitative results.

Coding was conducted independently by an experienced female nurse academic with expertise in qualitative research. Coding was undertaken manually, without the use of qualitative data analysis software. To enhance analytic rigour and transparency, an audit trail documenting code‐theme‐illustrative quotation linkages was maintained. A reflexive stance was adopted throughout the analysis, and the researchers repeatedly returned to the data to ensure that the themes remained grounded in participants' accounts.

Transcripts were returned to participants for verification. In reporting the findings, themes were supported by direct participant quotations, and participants were anonymised using codes (e.g., P1, P2…).

### 3.11 Mixed Methods Integration

Mixed methods integration was undertaken at the interpretation stage, in line with the logic of the explanatory sequential design (Creswell and Plano Clark 2018). Quantitative findings, including 5‐D pruritus severity categories and pruritus‐related variables, were integrated with the themes generated in the qualitative phase using a side‐by‐side joint display approach. Particular attention was paid to how the qualitative themes explained, expanded, or contextualised the quantitative findings. This process supported the development of clinically meaningful implications for routine symptom monitoring and nursing care based on the experiences of participants with moderate‐to‐severe pruritus.

### 3.12 Ethics

Ethical approval for the study was obtained from the relevant institutional review board. Written institutional permission was obtained from the administrations of the dialysis centres. All participants provided written informed consent after receiving verbal and written information about the study. Participation was voluntary, and participants were free to withdraw at any time without affecting their care. The study was conducted in accordance with the principles of the Declaration of Helsinki. Participant confidentiality was maintained by anonymising all study data.

### 3.13 Artificial Intelligence Use Statement

ChatGPT (OpenAI, GPT‐5.3) was used for language editing and manuscript refinement. It was not used for data generation, data analysis, or interpretation. The authors reviewed and approved all content and take full responsibility for the final manuscript.

## 4 Results

### 4.1 Quantitative Findings

According to Table 1, most patients receiving haemodialysis were female (61.6%), married (73.5%), and not employed (83.3%). The mean age was 58.9 ± 14.2 years. A large proportion of participants received haemodialysis three times per week (87.1%) and used an arteriovenous fistula (77.9%) as vascular access. In addition, 61.9% of participants had at least one comorbid chronic disease in addition to chronic kidney disease. Among the reported comorbidities, hypertension and diabetes mellitus were the most common.

**Table 1:** Sociodemographic and disease‐related characteristics of patients receiving haemodialysis (*n* = 294).

Variable	*n*	%
Sex		
Female	181	61.6
Male	113	38.4
Age; min–max: [58.9 ± 14.20; (18–74)]		
18–44	159	54.1
45–64	88	29.9
65–74	47	16.0
Educational level		
Literate (no formal diploma)	22	7.4
Primary school	99	33.7
High school	75	25.5
University	98	33.3
Marital status		
Married	216	73.5
Single	78	26.5
Employment status		
Employed	49	16.7
Not employed	245	83.3
Income status		
Income less than expenses	28	9.5
Income equal to expenses	232	78.9
Income greater than expenses	34	11.6
Duration of kidney disease		
1–5	85	28.9
5.1–10	70	23.8
10.1–15	58	19.7
15.1–20 years	81	27.6
Haemodialysis vintage		
1–5	43	14.6
5.1–10	102	34.7
10.1–15	74	25.2
15.1–20 years	75	25.5
Vascular access		
Arteriovenous fistula	229	77.9
Arteriovenous graft	5	1.7
Central venous catheter	60	20.4
Number of haemodialysis sessions/week^a^		
2 sessions	38	12.9
3 sessions	256	87.1
Presence of comorbid chronic disease (other than kidney failure)		
Yes	182	61.9
No	112	38.1
Type of comorbid chronic disease^b^		
Diabetes mellitus	86	29.3
Hypertension	99	33.7
Heart disease	16	5.4
Other	9	3.0

a No participant reported four haemodialysis sessions per week.

b Multiple responses were possible.

Most participants (72.1%) reported regular medication use for pruritus. The most commonly reported treatment types were oral antihistamines (48.6%) and creams/lotions (32.7%). Regarding treatment effect, 34.0% reported no effect, while 26.5% and 11.6% reported short‐term and long‐term benefit, respectively. Only 9.2% of participants reported receiving education on pruritus symptoms; among those who received education, 81.5% perceived it as adequate (Table 2).

**Table 2:** Distribution of pruritus and treatment‐related characteristics of patients receiving haemodialysis (*n* = 294).

Variable	*n*	%
Regular medication use for pruritus		
Yes	212	72.1
No	82	27.9
Type of medication used^a^		
Oral antihistamines	143	48.6
Creams/lotions	96	32.7
Duration of medication effect		
No effect	100	34.0
Short‐term effect (maximum 24 h)	78	26.5
Long‐term effect (> 24 h)	34	11.6
Received education on pruritus symptoms		
Yes	27	9.2
No	267	90.8
Perceived adequacy of education		
Adequate	22	81.5
Not adequate	5	18.5

a Multiple responses were possible for medication type.

The 5‐D Itch Scale total scores ranged from 5.00 to 19.75, with a mean score of 8.00 ± 3.21. When participants were categorised according to the severity classification proposed by Lai et al. (2017), 193 (65.6%) had no pruritus, 53 (18.0%) had mild pruritus, 37 (12.6%) had moderate pruritus, and 11 (3.7%) had severe pruritus. No participants were classified as having very severe pruritus. Accordingly, the prevalence of moderate‐to‐severe pruritus (moderate + severe categories) was 16.3% (*n* = 48). This group constituted the qualitative sampling frame for the second phase of the study (Table 3).

**Table 3:** Distribution of 5‐D Itch Scale scores and pruritus prevalence.

5‐D Itch scale variable		
5‐D Itch scale total score	Mean±SD = 8.00 ± 3.21; Min–Max: 5.00–19.75	
Severity category	*n*	%
No pruritus (5–8)	193	65.6
Mild pruritus (9–11)	53	18.0
Moderate pruritus (12–17)	37	12.6
Severe pruritus (18–21)	11	3.7
Very severe pruritus (≥ 22)	0	0.0
Any pruritus	101	34.4
Moderate‐to‐severe pruritus	48	16.3

Abbreviations: Max, maximum; Min, minumum; SD, standard deviation.

Table 4 compares the distribution of participants in the no/mild pruritus and moderate/severe pruritus groups (based on the 5‐D severity classification) across sociodemographic and clinical characteristics. No statistically significant between‐group differences were observed for sex (*χ* ^2^ = 0.021, *p* = 0.884), age group (*χ* ^2^ = 0.222, *p* = 0.895), educational level (*χ* ^2^ = 4.892, *p* = 0.180), employment status (*χ* ^2^ = 0.179, *p* = 0.672), income status (*χ* ^2^ = 1.719, *p* = 0.423), duration of kidney disease (*χ* ^2^ = 0.537, *p* = 0.911), HD vintage (*χ* ^2^ = 2.537, *p* = 0.469), vascular access type (*χ* ^2^ = 2.709, *p* = 0.100), or number of HD sessions per week (*χ* ^2^ = 1.166, *p* = 0.280).

**Table 4:** Prevalence of moderate‐to‐severe pruritus across sociodemographic and clinical characteristics (*n* = 294).

Variable	Category	No/mild pruritus *n* (%)	Moderate‐to‐severe pruritus *n* (%)	Total *n* (%)	*χ* ^2^	*p*
Sex	Female	151 (83.4)	30 (16.6)	181 (61.6)	0.021	0.884
	Male	95 (84.1)	18 (15.9)	113 (38.4)		
Marital status	Married	187 (86.6)	29 (13.4)	216 (73.5)	5.014	0.025
	Single	59 (75.6)	19 (24.4)	78 (26.5)		
Age group (years)	18–44	39 (83.0)	8 (17.0)	47 (16.0)	0.222	0.895
	45–64	75 (85.2)	13 (14.8)	88 (29.9)		
	65–74	132 (83.0)	27 (17.0)	159 (54.1)		
Educational level	Literate	16 (72.7)	6 (27.3)	22 (7.5)	4.892	0.180
	Primary school	86 (86.9)	13 (13.1)	99 (33.7)		
	High school	66 (88.0)	9 (12.0)	75 (25.5)		
	University and above	78 (79.6)	20 (20.4)	98 (33.3)		
Employment status	Employed	42 (85.7)	7 (14.3)	49 (16.7)	0.179	0.672
	Not employed	204 (83.3)	41 (16.7)	245 (83.3)		
Income status	Income expenses	21 (75.0)	7 (25.0)	28 (9.5)	1.719	0.423
	Income expenses	196 (84.5)	36 (15.5)	232 (78.9)		
	Income expenses	29 (85.3)	5 (14.7)	34 (11.6)		
Duration of kidney disease	1–5	71 (83.5)	14 (16.5)	85 (28.9)	0.537	0.911
	5.1–10	57 (81.4)	13 (18.6)	70 (23.8)		
	10.1–15	50 (86.2)	8 (13.8)	58 (19.7)		
	15.1–20 years	68 (84.0)	13 (16.0)	81 (27.6)		
HD vintage	1–5	33 (76.7)	10 (23.3)	43 (14.6)	2.537	0.469
	5.1–10	86 (84.3)	16 (15.7)	102 (34.7)		
	10.1–15	65 (87.8)	9 (12.2)	74 (25.2)		
	15.1–20 years	62 (82.7)	13 (17.3)	75 (25.5)		
Vascular access	AVF/graft	200 (85.5)	34 (14.5)	234 (79.6)	2.709	0.100
	Central venous catheter	46 (76.7)	14 (23.3)	60 (20.4)		
Number of HD sessions/week	2 sessions	29 (76.3)	9 (23.7)	38 (12.9)	1.166	0.280
	3 sessions	217 (84.8)	39 (15.2)	256 (87.1)		
Presence of comorbid chronic disease	Yes	146 (80.2)	36 (19.8)	182 (61.9)	4.171	0.041
	No	100 (89.3)	12 (10.7)	112 (38.1)		
Pruritus‐related education	Yes	18 (66.7)	9 (33.3)	27 (9.2)	6.295	0.012
	No	228 (85.4)	39 (14.6)	267 (90.8)		

In contrast, statistically significant differences were identified for marital status (*χ* ^2^ = 5.014, *p* = 0.025), presence of comorbid chronic disease (*χ* ^2^ = 4.171, *p* = 0.041), and receipt of pruritus‐related education (*χ* ^2^ = 6.295, *p* = 0.012). The proportion of participants with moderate/severe pruritus was higher among single participants than married participants (24.4% vs. 13.4%), among those with comorbid chronic disease than those without (19.8% vs. 10.7%), and among those who reported receiving pruritus‐related education than those who did not (33.3% vs. 14.6%).

Table 5 presents the results of the multivariable binary logistic regression analysis performed to identify factors independently associated with moderate‐to‐severe pruritus (defined as the moderate + severe categories of the 5‐D severity classification) in patients receiving haemodialysis. The model included sex, marital status, presence of comorbid chronic disease, age group, and receipt of pruritus‐related education. In the adjusted analysis, marital status, presence of comorbid chronic disease, and receipt of pruritus‐related education were significantly associated with moderate‐to‐severe pruritus.

**Table 5:** Factors associated with moderate‐to‐severe pruritus in patients receiving haemodialysis: multivariable binary logistic regression analysis.

Variable	Category (reference)	*B*	S.E.	Wald	*p*	OR (Exp(B))	95% CI (Lower)	95% CI (Upper)
Sex	Male vs. Female (ref)	0.027	0.345	0.006	0.938	1.027	0.522	2.021
Marital status	Married vs. Single (ref)	−0.872	0.372	5.497	0.019	0.418	0.202	0.867
Presence of comorbid chronic disease	Yes vs. No (ref)	0.875	0.386	5.136	0.023	2.399	1.126	5.112
Age group	18‐44 vs. 65–74 (ref)	−0.113	0.512	0.049	0.825	0.893	0.327	2.437
	45‐64 vs. 65–74 (ref)	−0.181	0.379	0.228	0.633	0.834	0.397	1.755
Pruritus‐related education	Yes vs. No (ref)	1.147	0.390	6.009	0.014	3.145	1.258	7.859
Constant	—	−1.713	0.495	11.971	0.001	0.180	—	—

Abbreviations: *B*, regression coefficient; CI, confidence interval; OR, odds ratio; S.E, standard error.

Being married (vs. single) was associated with lower odds of moderate‐to‐severe pruritus (*B* = −0.872, Wald = 5.497, *p* = 0.019, OR = 0.418, 95% CI 0.202–0.867). In contrast, the presence of at least one comorbid chronic disease was associated with higher odds of moderate‐to‐severe pruritus (*B* = 0.875, Wald = 5.136, *p* = 0.023, OR = 2.399, 95% CI 1.126–5.112). Similarly, participants who reported receiving pruritus‐related education had higher odds of moderate‐to‐severe pruritus than those who did not (*B* = 1.147, Wald = 6.009, *p* = 0.014, OR = 3.145, 95% CI 1.258–7.859). By contrast, sex (*p* = 0.938) and age group (18–44 vs. 65–74: *p* = 0.825; 45–64 vs. 65–74: *p* = 0.633) were not significantly associated with moderate‐to‐severe pruritus after adjustment.

### 4.2 Qualitative Findings

Reflexive thematic analysis showed (Table 6) that participants experienced pruritus as more than a clinical symptom; it was described as a multidimensional burden affecting sleep continuity, emotional wellbeing, social interactions, communication within the dialysis setting, and expectations of care. Four themes were developed: Pruritus Burden, Coping Mechanisms, Communication Barriers, and Care Expectations. Across participants' narratives, pruritus was linked to disrupted sleep, reduced life satisfaction, temporary and often insufficient coping attempts, perceived communication barriers in care encounters, and a clear expectation for more structured and individualised nursing care.

**Table 6:** Reflexive thematic analysis findings on pruritus experiences in patients receiving haemodialysis: themes, subthemes and representative participant quotations.

Themes	Subthemes	Representative participant quotations
Pruritus Burden	Disruption of sleep continuity	P‐01: “It starts in the evenings before dialysis, like needle‐pricking. It interrupts my sleep, ….” P‐31: “Night sleep is torture. I come to dialysis exhausted in the morning.”
	Reduced life satisfaction	P‐23: “There is no part of my body left that does not itch. I feel … emotionally collapsed.” P‐47: “My life energy has dropped. I no longer enjoy anything.”
	Perceived social stigma	P‐03: “I feel ashamed, as if people look at me like I am unclean.” P‐11: “My grandchild avoids me, thinking it is contagious….”
Coping Mechanisms	Self‐management attempts	P‐01: “I use a cold compress; it relieves a little, but only temporarily.” P‐32: “My own methods are temporary. I need a permanent treatment.”
	Limited benefit of pharmacological approaches	P‐06: “The medicine sometimes makes me drowsy, but it does not fully relieve it.” P‐15: “I use medication, but the side effects are too much… I feel groggy.”
	Risky practices related to lack of knowledge	P‐10: “I apply cologne… it ruins my skin.” P‐27: “Desperately, I put whatever I can find on my skin….”
Communication Barriers	Devaluation of the symptom	P‐01: “Because of the cliché ‘your phosphorus is high,’ I do not share it much.” P‐29: “When they say ‘it is normal,’ I feel devalued.”
	Busyness, hesitation, and suppressed symptom expression	P‐02: “Nurses are busy; I hesitate to mention it….” P‐36: “Sometimes I stay silent because they say there is nothing to do….”
Care Expectations	Expectation for monitoring pruritus	P‐21: “They should monitor itching as well [as other clinical issues].” P‐23: “They should examine our skin and document it at every session.”
	Expectation for individualised care	P‐01: “I want an effective, scientifically grounded skin care plan and follow‐up.” P‐24: “They should evaluate the causes and treatments of itching together with us….”

#### 4.2.1 Theme 1: Pruritus Burden

Participants described pruritus as a persistent and disruptive symptom that interfered with sleep continuity and daily functioning. Accounts also reflected emotional exhaustion, reduced enjoyment of life, and social stigma (e.g., shame, withdrawal, or concerns about how others perceived them). These narratives indicate that pruritus was experienced as a cumulative burden rather than an isolated physical complaint.

#### 4.2.2 Theme 2: Coping Mechanisms

Participants reported a range of self‐management attempts, including cooling strategies, emollient use, and personal routines; however, these were commonly described as providing only temporary relief. Pharmacological approaches were often perceived as only partially effective and sometimes accompanied by burdensome side effects (e.g., drowsiness/grogginess). Some participants also reported risky or unsuitable practices, suggesting a gap in practical symptom‐management guidance.

#### 4.2.3 Theme 3: Communication Barriers

Participants described difficulties in communicating pruritus in routine care, including experiences of symptom devaluation (e.g., being told it was “normal” or reduced to a single cause) and barriers related to busy care environments. Some narratives suggested that these interactions discouraged further disclosure and contributed to the symptom becoming less visible in clinical encounters.

#### 4.2.4 Theme 4: Care Expectations

Participants expressed clear expectations for pruritus to be taken seriously and monitored more systematically within dialysis care. They also expected more individualised, evidence‐informed care and education, including practical guidance, collaborative problem‐solving, and continuity in nursing approaches. In several accounts, participants additionally highlighted the need for supportive communication and family‐oriented information.

### 4.3 Mixed‐Methods Integration

When integrated, the quantitative and qualitative findings showed that pruritus in patients receiving haemodialysis is not only a symptom severity issue, but also a multidimensional care problem shaped by sleep disruption, psychosocial burden, communication barriers, and unmet support needs. Quantitatively, any pruritus was present in 34.4% of participants and moderate‐to‐severe pruritus in 16.3%; qualitatively, participants described disrupted sleep, reduced life satisfaction, stigma, temporary coping attempts, and difficulties expressing symptoms in routine care.

Regular medication use for pruritus was common, but treatment benefit was often limited, which was consistent with qualitative accounts of temporary relief and repeated self‐management attempts. The low proportion of participants receiving pruritus‐related education aligned with reports of risky practices and unmet support needs. In adjusted analysis, comorbid chronic disease and receipt of pruritus‐related education were associated with higher odds of moderate‐to‐severe pruritus, whereas being married was associated with lower odds. Qualitative findings suggest these associations should be interpreted within broader social and care contexts, and that the education finding likely reflects reactive care delivery rather than a harmful effect of education. Overall, the integrated findings support a structured approach to pruritus management in haemodialysis that includes routine monitoring, practical education, supportive communication, and individualised nursing care (Table 7).

**Table 7:** Concise joint display of quantitative and qualitative findings.

Quantitative finding	Related qualitative theme	Qualitative explanation	Integrated interpretation
Any pruritus prevalence was 34.4%; moderate‐to‐severe pruritus prevalence was 16.3%.	Pruritus Burden	Participants described sleep disruption, daytime fatigue, reduced life satisfaction, and perceived social stigma.	Quantitative prevalence shows that pruritus affects a substantial subgroup, while qualitative findings show that its impact extends beyond severity to functional and psychosocial burden.
Regular medication use for pruritus was high (72.1%).	Coping Mechanisms	Participants frequently reported medication use and self‐management attempts (e.g., cooling, skin care routines).	Many patients receiving haemodialysis actively try to manage pruritus, suggesting persistent symptom burden and ongoing care needs.
Treatment benefit was often limited (34.0% no effect; 26.5% short‐term effect).	Coping Mechanisms	Treatments were often described as temporary, insufficient.	Quantitative evidence of limited treatment effect is reinforced by qualitative accounts of temporary relief.
Only 9.2% reported receiving pruritus‐related education.	Coping Mechanisms/Care Expectations	Participants reported risky practices linked to lack of knowledge and expressed a need for practical, evidence‐informed guidance.	Low education coverage may contribute to trial‐and‐error coping and unsafe practices, highlighting the need for structured symptom education.
Comorbid chronic disease was associated with higher odds of moderate‐to‐severe pruritus (adj. OR = 2.399).	Pruritus Burden	Participants described cumulative burden (sleep disruption, emotional distress, daily life impact).	Comorbidity may represent a clinical context that increases vulnerability to more severe pruritus and/or greater perceived burden.
Being married was associated with lower odds of moderate‐to‐severe pruritus (adj. OR = 0.418).	Pruritus Burden/Communication Barriers	Narratives reflected stigma, isolation, and difficulties in sharing symptoms.	Marital status may reflect differences in social support and coping resources (interpretive inference).
Receipt of pruritus‐related education was associated with higher odds of moderate‐to‐severe pruritus (adj. OR = 3.145).	Care Expectations/Communication Barriers	Participants described delayed recognition, symptom devaluation, and expectations for earlier support.	This likely reflects reactive care delivery (patients with more severe symptoms are more likely to seek help and receive education), rather than a harmful effect of education.

## 5 Discussion

In this study, the quantitative phase characterised the prevalence and severity of chronic kidney disease‐associated pruritus in people receiving haemodialysis and indicated that moderate‐to‐severe pruritus affected a clinically meaningful subgroup. The observed prevalence was lower than that reported in some international haemodialysis cohorts; however, prevalence estimates vary substantially across studies due to differences in assessment instruments, recall periods, severity thresholds, and case definitions (Bai et al. 2025; Guedes et al. 2024; McMaster et al. 2025). In addition, the severity classification in this study followed the Five‐Dimensional Itch Scale categories proposed by Lai et al. (2017), which are clinically interpretable but not directly comparable with classifications derived from other instruments. A further quantitative finding was that pruritus treatment use was common, yet perceived benefit was often limited, suggesting that symptom control may remain suboptimal when pruritus is not systematically assessed and monitored in routine care (Latus et al. 2025).

The qualitative phase provided important insight into how pruritus is experienced in daily life and within care contexts. Participants described pruritus as a fluctuating and multidimensional burden disrupting sleep, diminishing life satisfaction, and shaping social experience, including stigma and communication challenges. Repeated self‐management attempts were commonly reported, although these often resulted in only temporary relief. These findings are consistent with European electronic patient‐reported outcome data indicating that the measured burden of pruritus and its associations with patient‐reported outcomes depend on the assessment instrument used, and they reinforce the importance of proactively eliciting symptoms in clinical practice (Guedes et al. 2024).

When findings were integrated, the association between comorbid chronic disease and higher odds of moderate‐to‐severe pruritus suggested that pruritus may cluster with broader clinical complexity and symptom burden. The association between pruritus‐related education and higher symptom severity is most plausibly explained by severity‐driven exposure to education, whereby patients with more pronounced symptoms are more likely to seek support and therefore receive education, rather than reflecting an adverse effect of education itself. This interpretation aligns with evidence that chronic kidney disease‐associated pruritus may be underreported and underdiagnosed unless symptom assessment is systematic and embedded in routine follow‐up (Guedes et al. 2024; McMaster et al. 2025).

Taken together, these findings support a structured and practice‐oriented approach to pruritus management in haemodialysis settings. Such an approach should complement pharmacological treatment with routine symptom assessment, consistent documentation, practical patient education, supportive communication, and individualised care planning, consistent with current recommendations recognising intractable pruritus as clinically important (Kidney Disease: Improving Global Outcomes KDIGO CKD Work Group 2024; Latus et al. 2025).

The integration of quantitative and qualitative findings further supports the need to embed structured pruritus assessment within person‐centred haemodialysis nursing care. Previous studies have shown that chronic kidney disease‐associated pruritus may be under‐recognised in routine haemodialysis care and that patients may not always report itching unless they are asked directly (Rayner et al. 2017; Lanot et al. 2023). Recent nursing‐focused evidence also highlights the role of nephrology nurses in identifying chronic kidney disease‐associated pruritus, monitoring symptom burden and facilitating communication between patients and the multidisciplinary team (Liossatou et al. 2025). In this context, structured assessment tools such as the Five‐D Itch Scale may help nurses move beyond informal questioning and support more consistent documentation, follow‐up and individualised care planning.

## 6 Implications for Clinical Practice

The findings of this study indicate that chronic kidney disease‐associated pruritus should be assessed routinely and systematically in haemodialysis care. Because some patients may normalise pruritus or may not report it unless directly asked, nurses should actively enquire about this symptom. The Five‐D Itch Scale may be used as a feasible tool to monitor pruritus severity, distribution and its impact on daily life.

Pruritus should be considered a multidimensional symptom rather than only a dermatological complaint. Nursing assessment should therefore include not only itch severity, but also sleep disturbance, fatigue, psychological wellbeing, daily functioning, coping strategies, perceived treatment benefit and education needs.

Patient education should be individualised, clear and practical. Education should address skin hydration, avoidance of potential triggers, appropriate use of prescribed treatments and prevention of harmful self‐care practices. Regular symptom monitoring, evaluation of treatment response and communication within the multidisciplinary team may support earlier recognition and more consistent management of pruritus.

Overall, integrating structured pruritus assessment and person‐centred nursing care into routine haemodialysis practice may strengthen symptom management and contribute to improved quality of life.

## 7 Conclusion

Chronic kidney disease‐associated pruritus remains a multidimensional and clinically significant symptom in haemodialysis care. Moderate‐to‐severe pruritus affects an important subgroup of patients and is associated with both clinical complexity and everyday burden. Routine structured assessment and person‐centred nursing care are essential to improve the recognition and management of pruritus. Integrating systematic symptom screening into haemodialysis practice may support earlier identification and more responsive symptom management.

## 8 Limitations

This study has some limitations. The quantitative phase was conducted in three dialysis centres, which may limit the generalisability of the findings. In addition, the cross‐sectional design does not allow causal inferences. Data were based on self‐reported measures and may be subject to reporting bias. The qualitative findings reflect the experiences of a specific group of participants and may vary across different populations.

Another limitation concerns the exclusion of participants with a documented psychiatric diagnosis or a clinically evident psychiatric condition that could compromise reliable self‐reporting, informed participation or completion of the qualitative interview. This criterion was applied to support the reliability of self‐reported symptom assessment and the consistency of qualitative interview data. However, mental health problems, particularly depression and anxiety, are common among people receiving haemodialysis and may influence symptom perception, coping behaviours and quality of life. Therefore, excluding such participants may have introduced selection bias and may limit the generalisability and transferability of the findings to the broader haemodialysis population. Future studies should include people with psychiatric comorbidities, where ethically and methodologically appropriate, to obtain findings that are more representative of the broader haemodialysis population encountered in routine clinical practice.

## Author Contributions

**Gülay Turgay:** principal project leader, conceived study, participated in design and coordination, analysed the data, read and approved the final manuscript. **Çiğdem Özdemir Eler:** participated in design and coordination, undertook interviews, analysed the data, helped to draft the manuscript, read and approved the final manuscript.

## Funding

The authors have nothing to report.

## Ethics Statement

The study protocol was reviewed and approved by the Başkent University Social Sciences, Humanities and Arts Research Board on 6 October 2025 (Approval No. 17162298.600‐293). Written institutional permission was obtained from the administrations of the participating dialysis centres. All participants provided written informed consent after receiving verbal and written information about the study. Participation was voluntary, and participants were free to withdraw at any time without affecting their care. The study was conducted in accordance with the principles of the Declaration of Helsinki, and participant confidentiality was maintained by anonymising the study data.

## Conflicts of Interest

The authors declare no conflicts of interest.

## Supporting information


Supporting File

## Data Availability

The data that support the findings of this study are available on request from the corresponding author. The data are not publicly available due to privacy or ethical restrictions.
