# Supplementary material for: Chronic Kidney Disease‐Associated Pruritus in Haemodialysis: A Mixed‐Methods Study of Symptom Burden and Patient Experience
Source: J Ren Care. 2026 Jul 29;52(3):e70075. doi: 10.1111/jorc.70075 (PMC13417541; doi:10.1111/jorc.70075)
Supplement: Supplementary file 1 — Supporting File [file JORC-52-0-s001.docx]

| Participant Code: | Participant Name-Surname: |
| --- | --- |
| Date: | Start Time: |
| Interviewer: | End Time: |

**Appendix 1. Semi-structured interview guide**

| **No** | **Topic** | **Main question** | **Probes / prompts** |
| --- | --- | --- | --- |
| 1 | Onset and trajectory of pruritus | When did you first notice your itching? How has it changed over time? | - At what times of day is it usually worse (e.g., daytime vs night-time)? - Does it change on dialysis days, or before/after dialysis sessions? - Which body areas are most affected? Has the distribution changed over time? - When you feel itchy, what do you notice physically (e.g., burning, tingling, stinging) and emotionally? |
| 2 | Impact on daily life, sleep, and social relationships | How does itching affect your daily life, sleep, and social relationships? | - What changes have you noticed in your sleep (falling asleep, staying asleep, waking up)? - What is most difficult for you in daily activities because of itching? - Does itching affect your relationships with family or others? If so, how? |
| 3 | Coping and self-management strategies | What do you do to cope with itching? Which strategies help, and which do not? | - What self-care methods do you use at home (e.g., moisturisers, bathing, cooling, clothing changes)? - Which treatments or suggestions from nurses or doctors have helped you, and which have not? - Are there any barriers that make it difficult to follow recommendations (e.g., side effects, cost, access, time)? - What do you think makes your itching worse (e.g., stress, heat, dry skin, diet/phosphate, after dialysis)? |
| 4 | Symptom disclosure and barriers to reporting | What has your experience been like when discussing itching with nurses or other health professionals? | - Do you feel comfortable telling nurses when you have itching? Why or why not? - Have there been times when you wanted to mention it but did not? What prevented you? - When you talk about itching, what responses do you usually receive? - What do nurses typically say or do about your itching, and is this support sufficient for you? |
| 5 | Expectations and suggestions regarding nursing care and pruritus management | What are your expectations regarding nursing care and the management of your itching? | - What has been most helpful so far? - What do you feel is missing, or what would you like to be done differently? - If a routine “itch screening and follow-up” system were implemented in the dialysis unit, what would it look like (e.g., how often to ask, how to record, who to inform)? |
| Closing | Closing question | Is there anything else you would like to add that I have not asked but you feel is important? |  |

*Note: Interviews were conducted in XXXXX.. Illustrative quotations were translated into English for publication and checked for accuracy and meaning consistency by the research team.*
